# Intravascular Placement of A Nasogastric Tube: Clinical Presentation and Surgical Management

**DOI:** 10.1002/lary.70773

**Published:** 2026-07-31

**Authors:** Matteo Alicandri‐Ciufelli, Daniele Marchioni, Alice Ruggieri, Samuele Baldelli, Lesley De Pietri

**Affiliations:** ^1^ University Hospital of Modena Modena Italy; ^2^ New Civil Hospital Sant'agostino‐Estense (NOCSAE) Baggiovara Italy

**Keywords:** case report, enteral feeding complications, head and neck, iatrogenic injury, nasogastric tube malposition

## Abstract

We report a rare case of inadvertent intravascular placement of a nasogastric tube following pharyngeal perforation, with extension through the internal jugular vein into the central venous system. Prompt radiological diagnosis and controlled surgical removal allowed successful management, highlighting the importance of early recognition and a multidisciplinary approach to this potentially life‐threatening complication.

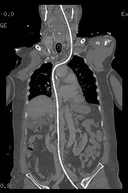

## 1 Introduction

Nasogastric tube (NGT) insertion is routinely performed for enteral feeding, gastric decompression, and medication delivery in both surgical and critical care settings. Although generally regarded as a low‐risk procedure, complications may occur, ranging from minor mucosal trauma to severe, life‐threatening events. The most commonly reported adverse events include pulmonary misplacement, aspiration, and esophageal perforation [1, 2] In contrast, severe vascular damage and/or intravascular misplacement of an NGT can be exceedingly rare, with no cases described in the literature to our knowledge. Such events could potentially result from pharyngeal or esophageal wall perforation with subsequent entry into adjacent vascular structures. A unique case of NGT misplacement involving cannulation of the internal jugular vein (IJV) and extensive progression through the venous system and heart is presented, highlighting diagnostic challenges and surgical management considerations relevant to otolaryngology practice.

## 2 Case Report

A 72‐year‐old man underwent cardiac valve replacement in another institution in February 2026. During the nocturnal post‐operative ICU stay, while still intubated, he had an NGT placement that was reported as “difficult” by the nursing staff. Soon after NGT positioning, blood was aspirated from it, and progressive anemia was noticed in the following hours. A chest x‐ray was performed, and malpositioning of the NGT was suspected. Therefore, he was transferred in the morning to our tertiary care referral center. A contrast‐enhanced total body CT‐scan showed an aberrant course of the NGT (Figure 1), that after entering the nasal cavity, perforated the oropharynx and parapharynx (Figure 2), cannulated the internal jugular vein, and coursed through the venous system via the superior vena cava, the right atrium, and the inferior vena cava. The intravascular course of the tube is further illustrated in Video 1. After consultation with the Otolaryngology team, emergency surgery was deemed necessary and agreed upon for NGT removal.

**FIGURE 1:**
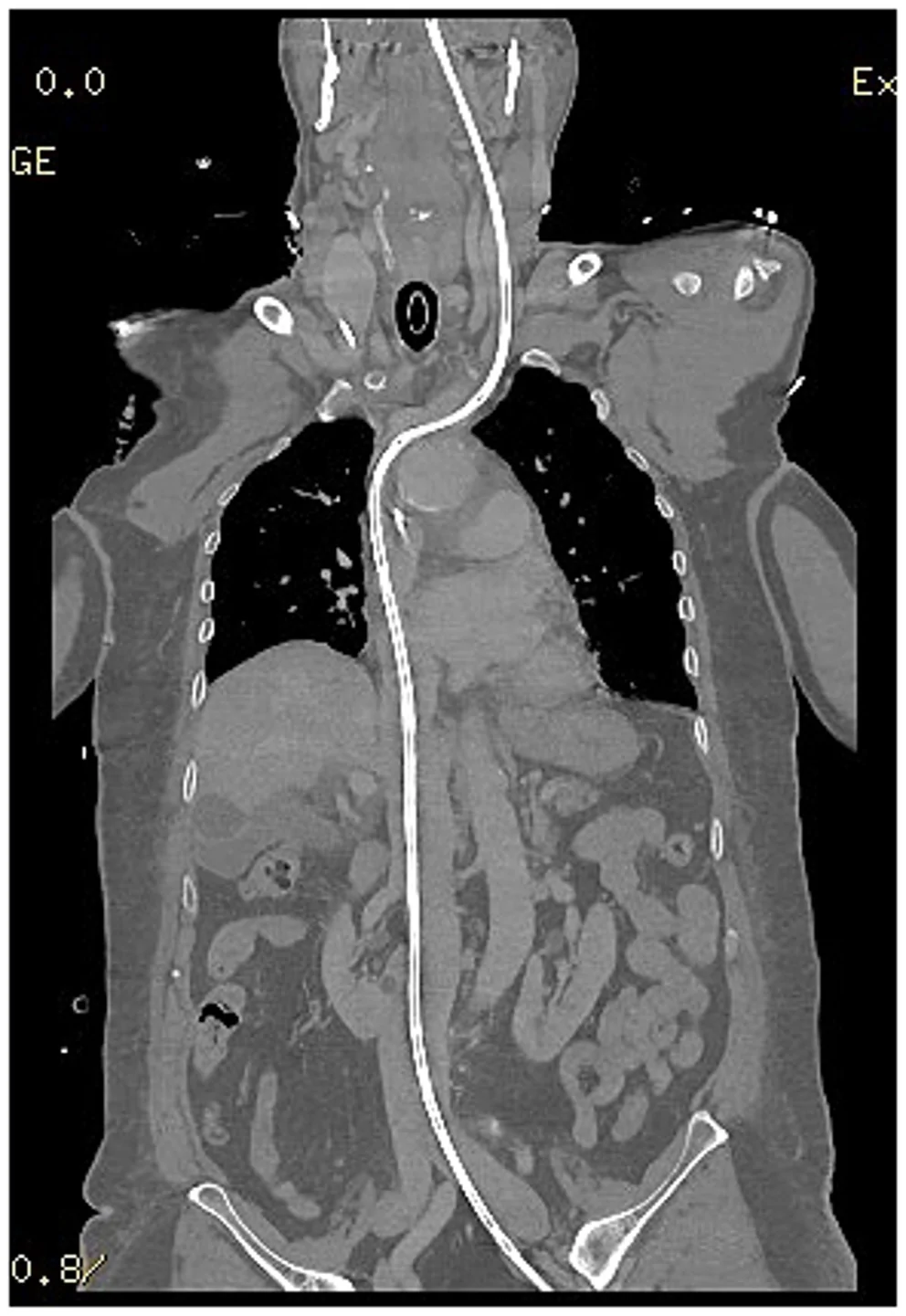
Curved planar reconstruction of total body contrast‐enhanced CT scan demonstrating the course of the nasogastric tube along the venous system.

**FIGURE 2:**
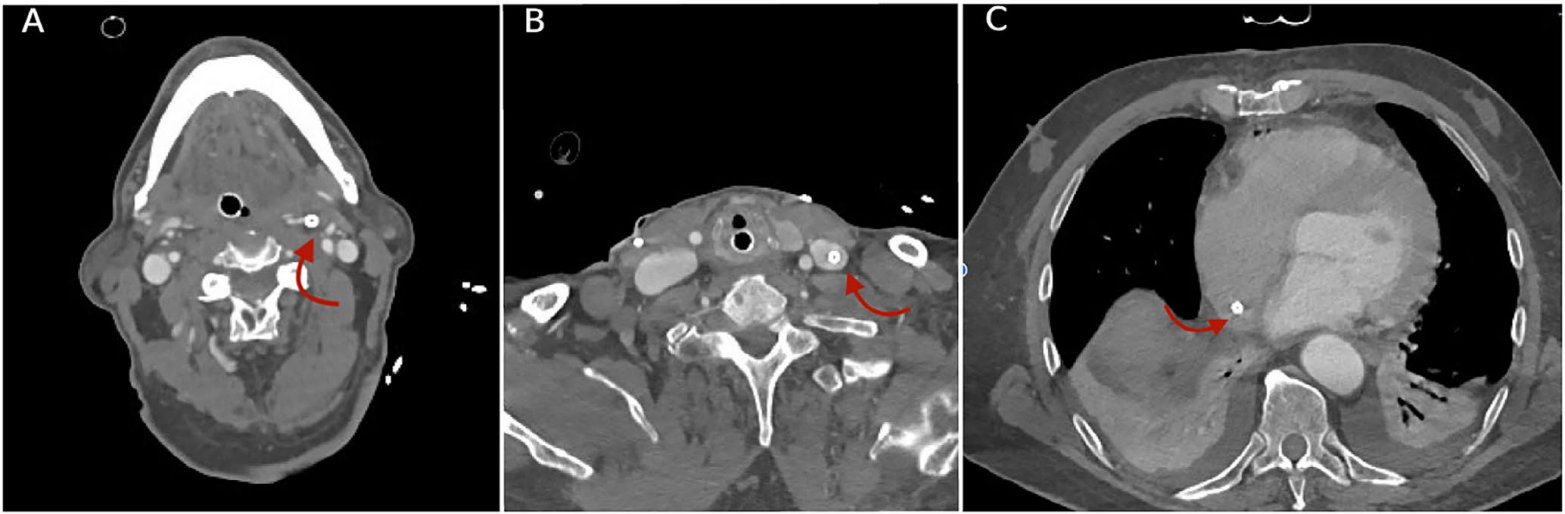
Panel A: Nasogastric tube entering in the parapharyngeal space (red arrow). Panel B: NGT in the right atrium (red arrow); Panel C: Nasogastric tube in the inferior vena cava (red arrow). [Color figure can be viewed in the online issue, which is available at www.laryngoscope.com]

**VIDEO 1:** Total body contrast‐enhanced axial CT scan showing the course of the nasogastric tube that, after entering the nasal cavity, perforated the oropharynx and parapharynx, cannulated the internal jugular vein, and coursed through the venous system via the superior vena cava, the right atrium, and the inferior vena cava, terminating in the left iliac vein. Video content can be viewed at https://onlinelibrary.wiley.com/doi/10.1002/lary.70773.

### 2.1 Surgical Treatment

A standard left cervicotomy was performed. The incision extended from the mastoid tip to the sternum. The sternocleidomastoid, digastric, stylohyoid, and infrahyoid muscles, as well as the submandibular gland and thyrolingual trunk, were identified and exposed. Careful dissection of the vascular bundle and internal jugular vein was carried out. No vascular injury was identified at this level; however, the NGT was clearly palpable within the internal jugular vein and could be followed superiorly toward the thyrolingual trunk and parapharyngeal space.

The anesthesiologist was asked to gradually withdraw the NGT until its distal end was palpated beyond the thyrolingual trunk, which was then safely ligated. Complete removal of the NGT was subsequently performed under direct monitoring.

Immediately after removal, significant bleeding from the oral cavity occurred. The surgical field was therefore extended to the parapharyngeal space, with sectioning of the digastric and stylohyoid muscles. Venous bleeding from the pterygoid plexus was identified and controlled with cauterization. Bleeding ceased both from the oral cavity and the neck.

A small defect in the lateral pharyngeal wall was identified and left unsutured due to its limited size and difficult accessibility. The parapharyngeal space was packed with resorbable hemostatic agents (Tabotamp and Spongostan). The wound was closed with placement of a drain, which was removed on postoperative Day 2. The postoperative course was uneventful, and oral feeding was resumed on postoperative Day 7. Postoperatively, the patient received intravenous antibiotic therapy with piperacillin/tazobactam (4.5 g every 6 h for 3 days) in combination with daptomycin (850 mg once daily for 2 days).

## 3 Discussion

Although NGT placement is considered a basic clinical skill, this case emphasizes that severe complications may arise. The present report describes an unusual intravascular trajectory of an NGT following perforation of the lateral oropharyngeal wall, with entry into the parapharyngeal space and into the IJV by pterygoid plexus veins, with a distal progression to the iliac venous system. To our knowledge, such an extensive intravascular course remains exceptionally rare, if not unique in the literature.

The lateral oropharyngeal wall represents a critical anatomical region where mucosal integrity is essential to prevent injury to adjacent neurovascular structures. Parapahryngeal space, with pterygoid venous plexus, and the carotid sheath, containing the IJV and carotid artery, lie in close proximity, rendering them vulnerable in the setting of traumatic perforations. Factors such as recent surgery, tissue edema, and altered anatomy further increase susceptibility to iatrogenic perforation [3].

The mechanism of injury in most reported cases involves forceful advancement of the tube despite resistance. This technical error can result in mucosal disruption and deviation from the intended esophageal pathway. Importantly, resistance during insertion should always prompt cessation and reassessment, as it may indicate coiling, obstruction, or misdirection. This principle is particularly relevant in sedated or intubated patients, where protective reflexes are diminished.

Clinical manifestations of intravascular NGT placement may be subtle. Typical warning signs described in the literature include failure to aspirate gastric contents, persistent coughing, or abnormal tube positioning on imaging. In the present case, progressive anemia and bleeding from the tube were key findings. Radiographic confirmation by chest X‐ray remains the gold standard for verifying proper placement while in case of suspected complications, a chest CT scan should be advised [4, 5].

Management of intravascular NGT misplacement requires careful planning. Blind removal should be contraindicated due to the risk of massive hemorrhage, air embolism, or vascular injury. Instead, controlled surgical or endovascular approaches should be recommended. In this case, a left cervicotomy provided direct access to the IJV, enabling safe extraction of the tube and ligation of the thyrolingual trunk, reducing the retrograde bleeding. Then an approach to the parapharyngeal space was prepared, having the digastric and stylohyoid muscles under direct view, and was then performed when after removing the NGT the bleeding occurred from the oropharynx. This approach ensured vascular control to the veins of the pterygoid plexus, from which eventually the NGT entered to the venous system.

From an anesthesiology and ICU staff perspective, this case highlights the importance of detailed anatomical knowledge and vigilance during upper aerodigestive tract traumatic procedures. Otolaryngologists should be aware of the potential for rare but severe complications and adopt a cautious and gradual surgical approach to manage massive bleeding during the procedure.

## 4 Conclusion

Intravascular misplacement of an NGT is a rare but serious complication associated with potential significant morbidity. Awareness of regional anatomy, avoidance of forceful insertion, and radiologic confirmation are essential preventive measures. Prompt recognition and controlled surgical management are critical to achieving favorable outcomes.

## Funding

The authors have nothing to report.

## Ethics Statement

The authors have nothing to report.

## Consent

Written informed consent was obtained from the patient for publication.

## Conflicts of Interest

The authors declare no conflicts of interest.

## Data Availability

The data that support the findings of this study are available on request from the corresponding author. The data are not publicly available due to privacy or ethical restrictions.
